# Sustained-Release Solid Dispersions of Fenofibrate for Simultaneous Enhancement of the Extent and Duration of Drug Exposure

**DOI:** 10.3390/pharmaceutics16121617

**Published:** 2024-12-20

**Authors:** Seong-Jin Park, Gyu Lin Kim, Hyo-Kyung Han

**Affiliations:** College of Pharmacy, Dongguk University-Seoul, Dongguk-ro-32, Ilsan-Donggu, Goyang 10326, Republic of Korea

**Keywords:** hydrophilic polymer, solubilization, dissolution, poloxamer 407, Eudragit^®^ RSPO, bioavailability

## Abstract

Background/Objectives: A sustained-release formulation of fenofibrate while enhancing drug dissolution with minimal food effect is critical for maximizing the therapeutic benefits of fenofibrate. Therefore, this study aimed to develop an effective solid dispersion formulation of fenofibrate for simultaneous enhancement in the extent and duration of drug exposure. Methods: Fenofibrate-loaded solid dispersions (FNSDs) were prepared using poloxamer 407 and Eudragit^®^ RSPO at varied ratios via solvent evaporation. In vitro/in vivo characteristics of FNSDs were examined in comparison with untreated drugs. Results: Based on dissolution profiles of FNSDs in aqueous media, the weight ratio of fenofibrate: poloxamer 407: Eudragit^®^ RSPO at 1:1:4 (FNSD2) was selected as the optimal composition for achieving sustained drug release while maximizing the drug dissolution. The enhanced and sustained drug release of FNSD2 was also confirmed in a buffer transition system mimicking the pH change in the gastrointestinal tract. FNSD2 achieved approximately 66% drug release over 12 h, while pure drug exhibited only 12%. Furthermore, FNSD2 maintained similar release rates under fed and fasted conditions, while the entire drug dissolution slightly increased in the fed state. Structural analysis by x-ray diffraction showed that fenofibrate remained crystalline in FNSD2. Pharmacokinetic studies in rats revealed that orally administered FNSD2 significantly improved the extent and duration of systemic drug exposure. Compared to pure drugs, the FNSD2 formulation increased the oral bioavailability of fenofibrate by 22 folds with the delayed T_max_ of 4 h in rats. Conclusion: FNSD2 formulation is effective in improving the extent and duration of drug exposure simultaneously.

## 1. Introduction

Cardiovascular diseases (CVDs) are among the leading causes of mortality worldwide. Elevated blood lipid levels are associated with a high risk of CVDs, making the prevention and effective management of dyslipidemia essential for enhancing the quality of life and promoting healthy longevity [1]. Fenofibrate, a peroxisome proliferator-activated receptor (PPAR)-α agonist, is used to treat patients with severe hypertriglyceridemia, primary hypercholesterolemia, or mixed dyslipidemia [2,3,4]. As a potent lipid regulator, fenofibrate lowers triglycerides, apolipoprotein B, and low-density lipoprotein cholesterol while increasing high-density lipoprotein cholesterol [2,3,4]. However, the oral absorption of fenofibrate is limited due to its low solubility (<0.4 μg/mL, classified as Biopharmaceutics Classification System Class II), and its bioavailability is significantly affected by food intake [5,6]. To overcome this limitation, various formulation techniques, including micronization, nanoparticles, self-microemulsifying drug delivery systems, and solid dispersions, have been employed to improve the solubility and oral bioavailability of fenofibrate [2,6,7]. For example, a novel formulation containing fenofibrate nanoparticles (Tricor^®^ 145 mg) and IDD-P (insoluble drug delivery-microparticle) fenofibrate tablets have been developed to improve the bioavailability of fenofibrate without the effect of food [8,9]. Recently, Kim et al. [10] reported that a supercritical fluid-assisted spray-drying process incorporating surface-active additives can efficiently micronize fenofibrate particles, improving drug dissolution. Alshamsan et al. [7] also developed the self-nanoemulsifying formulation of fenofibrate using blended oils (Kollisolv MCT and Imwitor 742) and significantly improved the solubility and dissolution of fenofibrate. Although there was great success in improving the dissolution and bioavailability of fenofibrate via various formulations, most commercially available formulations of fenofibrate, with a few exceptions, are still recommended for use with a meal [2]. Accordingly, there is a need to develop a new formulation to maximize the therapeutic benefits of fenofibrate. Particularly, a sustained-release formulation of fenofibrate while enhancing drug dissolution with minimal effects of food is crucial for enhancing oral bioavailability and reducing dosing frequency [9], ultimately improving patient compliance and offering additional benefits, such as cost savings and fewer side effects [6,11,12].

Among various formulation approaches, SDs within an insoluble polymer matrix provide a simple and effective technique for developing sustained-release oral dosage forms [13]. In SDs, the drug is uniformly distributed within the polymer matrix, with the release rate controlled by the polymer’s swelling or erosion [13,14]. A wide variety of hydrophilic and hydrophobic polymers are available commercially for SDs, each displaying distinct physicochemical properties. In preparing SDs, polymers are used alone or in combination with others to modulate the matrix’s permeability, thereby optimizing drug release rates. Polymethacrylate-based copolymers, derived from esters of acrylic and methacrylic acids, are among the most commonly used insoluble polymers for sustained drug release due to their high chemical stability, miscibility, compressibility, and versatility in diffusion control [15]. Specifically, Eudragit^®^ RSPO, a poly(ethyl acrylate-co-methyl methacrylate-co-trimethylammonioethyl methacrylate chloride) 1:2:0.1, is insoluble with a pH-independent swelling, and thus it is suitable for sustained drug release across varied gastrointestinal pH [16,17,18]. In addition, given fenofibrate’s low solubility and resulting limited dissolution in intestinal fluids, enhancing its solubility and achieving controlled release are necessary to improve oral bioavailability and extend drug exposure. In this context, adding a solubilizer to the hydrophobic polymer matrix of SDs is advantageous. Poloxamer 407 is a hydrophilic and non-ionic surfactant. It is biocompatible, non-toxic, and has low immunogenicity [19,20,21]. Consequently, poloxamer 407 is widely used as a solubilizer and wetting agent across various drug delivery systems, including oral, parenteral, ocular, and rectal routes [19,20,21]. Taken together, poloxamer 407 and Eudragit^®^ RSPO were selected to prepare solid dispersions of fenofibrate for not only enhancing drug dissolution rate but also sustaining drug release.

In this study, SDs were prepared using a solvent evaporation method with the blended mixture of poloxamer 407 and Eudragit^®^ RSPO, and the in vitro and in vivo effectiveness of the developed SDs was evaluated.

## 2. Materials and Methods

### 2.1. Materials

Fenofibrate, ketoprofen, poloxamer 407, and sodium taurocholate were obtained from Sigma-Aldrich (St. Louis, MO, USA). Poly(ethyl acrylate-co-methyl methacrylate-co-trimethylammonioethyl methacrylate chloride) 1:2:0.1 (Eudragit^®^ RSPO) powder was provided by Evonik Korea Ltd. (Seoul, Republic of Korea). Lecithin (soybean) was obtained from Daejung Chemicals & Metals Co., Ltd. (Siheung, Republic of Korea), while sodium oleate, inorganic salts, and polyethylene glycol sorbitan monooleate (Tween^®^80) were acquired from Junsei Chemical Co., Ltd. (Tokyo, Japan). Glyceryl monooleate was sourced from ABITEC Corporation (Columbus, OH, USA). All other chemicals were of analytical grade, and solvents, including acetonitrile and methanol, were HPLC grade.

### 2.2. Preparation of Fenofibrate-Loaded Solid Dispersions (FNSDs)

Since the amount of polymer is closely related to the dissolution efficiency of solid dispersions, FNSDs were prepared at various drug–polymer ratios via a solvent evaporation method. Based on the preliminary studies, the drug-to-polymer ratios were selected as outlined in Table 1. All formulation components (fenofibrate, Eudragit^®^ RSPO, and poloxamer 407) were dissolved in methanol and mixed thoroughly by stirring at 500 rpm for 15 min at room temperature. After the solvent was removed using a rotary evaporator, the resulting dispersions were air-dried under vacuum at 25 °C for 24 h. The obtained SDs (FNSD1–FNSD6) were ground and passed through a 60-mesh (250 μm) sieve. Physical mixtures (PMs) were also prepared by blending fenofibrate, Eudragit^®^ RSPO, and poloxamer 407 using a mortar and pestle.

The solubility of fenofibrate from different formulations (untreated powder, PMs, and FNSDs) was assessed in water. Each formulation containing 2 mg of fenofibrate was added to 1 mL of distilled water and incubated in a shaking water bath at 37 °C and 100 rpm for 48 h. After filtration through a 0.45 μm membrane, drug concentrations were determined by HPLC.

### 2.3. In Vitro Dissolution Study

Dissolution studies were carried out with a dissolution apparatus (DT 1420; ERWEKA GmbH, Heusenstamm, Germany) using the paddle method at an agitation speed of 50 rpm and a temperature of 37 ± 0.5 °C [22]. Each formulation (FNSD1 to FNSD6), loaded with 40 mg of fenofibrate, was encapsulated in hard gelatin capsules and immersed in water with 2% Tween 80. At specific time points, 1 mL samples were withdrawn, and a fresh medium was added to keep the volume consistent. The samples were filtered through a PTFE membrane filter (pore size: 0.45 μm) and analyzed using HPLC to determine the amount of drug released.

The drug release profiles of the optimized FNSD, PM, and pure drug were also assessed in a buffer transition system that mimics pH variations in the gastrointestinal (GI) tract. Each formulation equivalent to 40 mg of fenofibrate was exposed to 750 mL of 0.1 N hydrochloric acid solution containing 2% Tween 80 for 2 h. Then, the pH of the dissolution medium was adjusted to 6.8 by adding 0.3 M of tribasic sodium phosphate solution (143 mL) and 0.1 N of hydrochloric acid solution (7 mL). At specific intervals, 1 mL samples were collected, and a fresh dissolution medium was added to maintain the volume constant. The samples were filtered through a 0.45 μm PTFE membrane, and the drug concentration was measured using HPLC.

Different kinetic models, including the Higuchi and Korsmeyer–Peppas equations, were used to examine the dissolution kinetics of the optimized FNSD formulation [23,24,25].

### 2.4. Dissolution Studies in Simulated Intestinal Fluids

The impact of food intake on the dissolution profiles of the FNSD2 formulation and pure drug (untreated powder) was examined using simulated intestinal fluids reflecting fasted (FaSSIF) and fed state (FeSSIF) [26]. The compositions of FaSSIF and FeSSIF are as follows: (i) FaSSIF: sodium taurocholate (3 mM), lecithin (0.2 mM), maleic acid (19.12 mM), sodium hydroxide (34.8 mM), and sodium chloride (68.62 mM), adjusted to pH 6.5, and (ii) FeSSIF: sodium taurocholate (10 mM), lecithin (3 mM), maleic acid (28.6 mM), sodium hydroxide (52.5 mM), sodium chloride (145.2 mM), glyceryl monooleate (6.5 mM), and sodium oleate (40 mM), adjusted to pH 6.5. Dissolution studies were conducted at a paddle speed of 50 rpm and a temperature of 37 ± 0.5 °C under sink conditions with 2% Tween 80, following the procedure described in Section 2.3. Released drug amounts were determined via HPLC.

### 2.5. Structural Characterization

#### 2.5.1. X-Ray Diffraction (XRD)

XRD analysis was performed using an X-ray diffractometer (X’Pert Pro MRD; Malvern Panalytical Ltd., Malvern, UK). Diffraction patterns were obtained with CuKα radiation at a scan rate of 2 s per step. The analysis was performed at the Korea Basic Science Institute (Daegu, Republic of Korea).

#### 2.5.2. Differential Scanning Calorimetry (DSC) Analysis

Thermal transition properties of each formulation component, PM, and FNSD were analyzed using a DSC (DSC Q2000; TA Instruments, New Castle, DE, USA). Calibration for temperature and heat flow was performed using indium. Samples were placed in aluminum pans, and thermograms were recorded within a temperature range from 0 °C to 300 °C at a scanning rate of 10 °C/min under a nitrogen atmosphere with a flow rate of 30 mL/min.

### 2.6. Pharmacokinetic Studies in Rats

Pharmacokinetic profiles of fenofibrate were assessed in rats following oral administration of fenofibrate (untreated powder), PM, and the optimized FNSD formulation. The experimental protocol was approved by the Institutional Animal Care and Use Committee of Dongguk University (IACUC-2023-038-01, 24 January 2024). Male Sprague-Dawley rats (250–280 g) were provided by Orient Bio Inc. (Seongnam, Republic of Korea). Prior to the experiment, the rats were fasted for 12 h and divided into three groups (n = 6/group). Each group received an oral dose of fenofibrate (20 mg/kg) in one of the following formulations: FNSD2, PM, or pure drug. Each formulation was dispersed in 0.5% aqueous methylcellulose. Blood samples were collected from the jugular vein at specified time points, centrifuged at 22,250× *g* for 5 min at 4 °C, and the plasma samples were stored at −20 °C until analyzed by HPLC.

### 2.7. HPLC Assay

#### 2.7.1. In Vitro Samples

Fenofibrate concentrations were measured with an HPLC system (Flexar, PerkinElmer, Shelton, CT, USA). The mobile phase of acetonitrile: water (80:20, *v*/*v*, pH 3.5) was eluted from a reversed-phase C18 column (Gemini C18, 4.6 × 250 mm, 5 μm; Phenomenex, Torrance, CA, USA) at a flow rate of 1 mL/min. The UV detection wavelength was set at 254 nm [27], and the calibration curve for fenofibrate was linear over a concentration range of 0.1–100 μg/mL (r^2^ = 0.999).

#### 2.7.2. In Vivo Samples

Plasma concentrations of fenofibric acid, a metabolite of fenofibrate, were determined using a reversed-phase C18 column (Gemini C18, 4.6 × 250 mm, 5 μm; Phenomenex, Torrance, CA, USA) at 40 °C. The mobile phase was composed of acetonitrile:0.2% phosphoric acid (50:50, *v*/*v*) and eluted from the column at a flow rate of 1 mL/min. The UV detection wavelength was set at 286 nm. Plasma samples (100 μL) were mixed with 20 μL of internal standard (100 μg/mL ketoprofen), followed by 180 μL of acetonitrile with vigorous mixing. The mixture was centrifuged at 25,000× *g* for 10 min and the drug concentration in the supernatant was determined by HPLC. Calibration curves were linear from 1 to 100 μg/mL (r^2^ = 0.999).

### 2.8. Pharmacokinetic and Statistical Data Analysis

The pharmacokinetic data analysis was performed using the WinNonlin^®^ 2.1 software (Certara, Radnor, PA, USA). The maximum plasma concentration (C_max_) and the time to reach C_max_ (T_max_) were determined by visually inspecting plasma concentration-time profiles. The linear trapezoidal method was used to estimate the area under the plasma concentration-time curve (AUC).

Data are presented as mean ± standard deviation. Statistical analysis was performed using Student’s *t*-test or ANOVA with a *p*-value less than 0.05, indicating statistical significance.

## 3. Results and Discussion

### 3.1. Fabrication and Characterization of Sustained-Release Solid Dispersions

**Figure 1:**
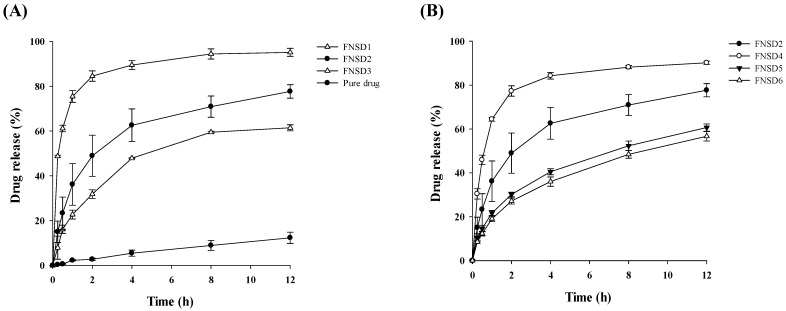
Effect of the formulation compositions on the dissolution profiles of fenofibrate in water with 2% Tween 80 (Mean ± SD, n = 3). (**A**) Effect of poloxamer 407; (**B**) effect of Eudragit^®^ RSPO.

Fenofibrate-loaded solid dispersions (FNSDs) were fabricated using a blend of poloxamer 407 and Eudragit^®^ RSPO to achieve sustained drug release and enhanced solubility of fenofibrate. FNSDs with varying drug-to-polymer ratios were prepared to optimize formulation composition, as shown in Table 1. Results demonstrated that the dissolution of all FNSD formulations (FNSD1–FNSD6) was significantly improved compared to pure drug powder that exhibited only 12.3% drug release over 12 h. Depending on drug-to-polymer ratios, FNSD formulations exhibited drug release between 53.5% and 95.2% (Figure 1).

When the weight ratio of the drug to poloxamer 407 increased from 1:0.5 to 1:2, drug dissolution improved from 31.9% to 84.6% after 2 h and from 61.5% to 95.2% over 12 h (Figure 1A). In parallel, FNSDs significantly increased drug solubility as the ratio of poloxamer 407 increased. As shown in Table 2, fenofibrate has poor aqueous solubility (0.21 ± 0.05 μg/mL), which is comparable to the result previously reported [22]. In contrast, FNSDs increased the solubility of fenofibrate by 1.7–57 folds, depending on the ratio of poloxamer 407. Consequently, FNSDs having a greater portion of poloxamer 407, such as FNSD3, displayed immediate drug release characteristics with rapid and nearly complete drug dissolution (Figure 1A). These results highlight the effect of poloxamer 407 in reducing interfacial tension and improving drug wettability in FNSD formulations where drugs are uniformly distributed into the polymer matrix [20]. In addition to the solubilization effect of poloxamer 407, SD formulations may have additional advantages, including particle size reduction and change of drug crystallinity [15]. As a result, solubility enhancement was much higher in FNSDs compared to those in the corresponding PMs (Table 2). Overall, poloxamer 407, a hydrophilic non-ionic surfactant, improved the drug solubility and accelerated drug release from the polymeric matrix as its concentration increased.

Conversely, increasing the proportion of Eudragit^®^ RSPO from 1:2 to 1:10 reduced drug release from 77.3% to 27.1% at 2 h and from 90.2% to 53.5% at 12 h (Figure 1B). Eudragit^®^ RSPO, a hydrophobic polymer, formed a viscous barrier upon swelling, which delayed water penetration and drug diffusion [28]. In FNSDs, Eudragit^®^ RSPO could act as a rate-controlling carrier, while Poloxamer 407 acted as a drug release stimulant and provided an environment to promote swelling of Eudragit^®^ RSPO. Therefore, the combined use of Eudragit^®^ RSPO and poloxamer 407 could afford FNSDs with sustained drug release behavior while enhancing cumulative drug dissolution.

According to the FIP (International Pharmaceutical Federation) guideline and European Pharmacopeia for sustained release formulations, the first specification sets around 20–30% drug release after one or two hours to prevent dose dumping, while the cumulative drug dissolution at the last time point should be at least 80% to ensure almost quantitative release [29]. While FNSD3 and FNSD4 exhibited fast drug release profiles, the other four formulations (FNSD1, FNSD2, FNSD5, and FNSD6) showed a drug release of 20–35% in 1 h, preventing premature drug release. Among these formulations, FNSD2 exhibited the highest drug dissolution (approximately 78%) at 12 h, while the others (FNSD1, FNSD5, and FNSD6) exhibited less than 60%. As the dissolution profile of FNSD2 met the criteria for sustained release formulations, FNSD2 was selected as an optimal formulation.

Drug release kinetics for FNSD2 were further assessed using mathematical models. As summarized in Table 3, the drug release of FNSD2 showed a good correlation for all the models except the zero order. FNSD2 follows first-order kinetics, where drug release is concentration-dependent, and the released drug amount decreases with decreasing concentration gradient over time [24]. It also fits well with the Higuchi equation (r^2^ = 0.9263), indicating a diffusion-controlled release [24]. Furthermore, the drug dissolution of FNSD2 is best fitted to Korsmeyer–Peppas with the highest correlation (r^2^ = 0.9552) and the release exponent (n) of 0.407. This result suggests that FNSD2 follows the Fickian diffusion-controlled drug release [24].

**Figure 2:**
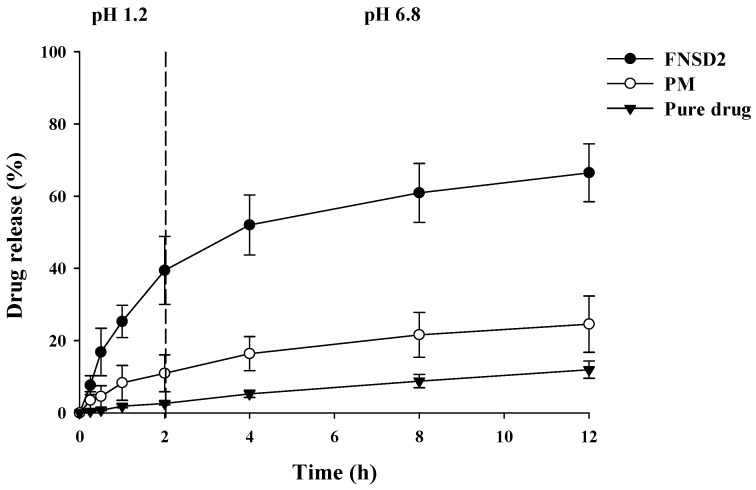
Dissolution profiles of fenofibrate from different formulations in a buffer transition system (Mean ± SD, n = 6).

The dissolution characteristics of FNSD2 were also evaluated in a buffer transition system mimicking gastrointestinal pH. Compared to pure drug powder and PM, FNSD2 dramatically enhanced the drug dissolution while sustaining the drug release, as shown in Figure 2. FNSD2 achieved a 39.4% release at pH 1.2 after 2 h, followed by a gradual release to 66.5% over 12 h at pH 6.8. Since Eudragit^®^ RSPO swells pH independently [16], gradual drug release from FNSD2 was achieved over the pH range from 1.2 to 6.8.

### 3.2. Dissolution Studies in Simulated Intestinal Fluids

Taking food with fenofibrate has a positive impact on drug absorption since the increased bile salt concentration can promote drug dissolution in the GI tract via micellar formation [5,6,8]. As a result, fenofibrate has variable fed and fasted bioavailability, and its oral intake with a meal, especially a high-fat diet, is beneficial for maximizing drug absorption [6,30]. However, in current medical practice, fenofibrate is often used in combination with statins for patients with hyperlipidemia who should strictly adhere to a low-fat diet [31,32]. Therefore, a formulation with minimal food effect is demanded to improve the clinical benefits of fenofibrate [2,6].

**Figure 3:**
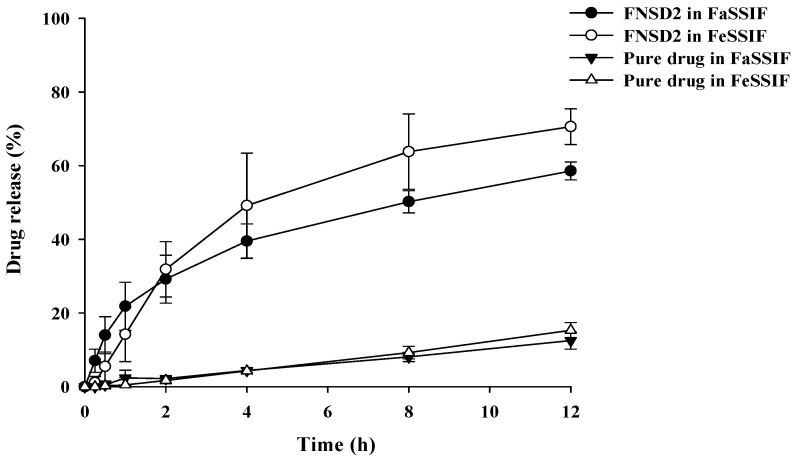
Dissolution profiles of fenofibrate and the FNSD2 formulation in fasted (FaSSIF) and fed (FeSSIF) conditions (Mean ± SD, n = 6).

In the present study, the potential effect of food on the dissolution of FNSD2 and pure drug was evaluated using simulated intestinal fluids (FaSSIF and FeSSIF) reflecting fasted and fed conditions. In both pure drug and FNSD2, drug dissolution tends to be increased in the fed condition compared to those in the fasted condition (Figure 3). These results are attributed to the higher concentrations of bile salt and lipid digestion products such as glyceryl monooleate and sodium oleate in FeSSIF. Since bile salt and lipid digestion products act as bio-surfactants and enhance the solubility and dissolution of lipophilic drugs via micellar formation, their presence in higher concentrations increased drug dissolution to a greater extent in fed conditions [33]. Since FNSD2 achieved high drug dissolution in both fed and fasted conditions, it may minimize the effect of food intake on drug absorption. Furthermore, the FNSD2 formulation maintained the sustained drug release profile regardless of food intake.

### 3.3. Structural Analysis

**Figure 4:**
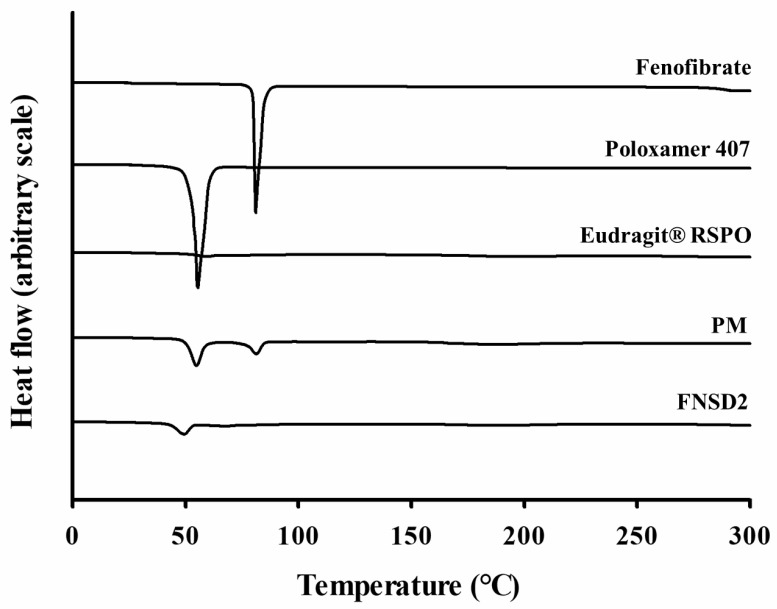
DSC thermograms of fenofibrate, poloxamer 407, Eudragit^®^ RSPO, physical mixture (PM), and the FNSD2 formulation.

Thermal properties of each formulation component (drug and excipients), PM, and FNSD2 were examined using DSC. Due to the regular arrangement of atoms, crystalline compounds melt at once, displaying a sharp peak in DSC [34]. While Eudragit^®^ RSPO was amorphous, pure drug and poloxamer 407 exhibited a sharp endothermic peak at 81.2 °C and 55.5 °C, respectively, indicating they are in crystalline structures (Figure 4). These peaks were retained in the PM, implying that the physical mixing of the drug and excipients does not alter the crystalline states, and the excipients are compatible with the drug. In contrast, the endothermic peak of fenofibrate disappeared in the FNSD2 formulation. This result may be because (i) the crystalline state of the drug was changed to an amorphous state or (ii) drugs were completely dissolved in the molten carrier to produce a single homogeneous phase in which the drug and the polymers are intimately mixed at a molecular level [35,36].

**Figure 5:**
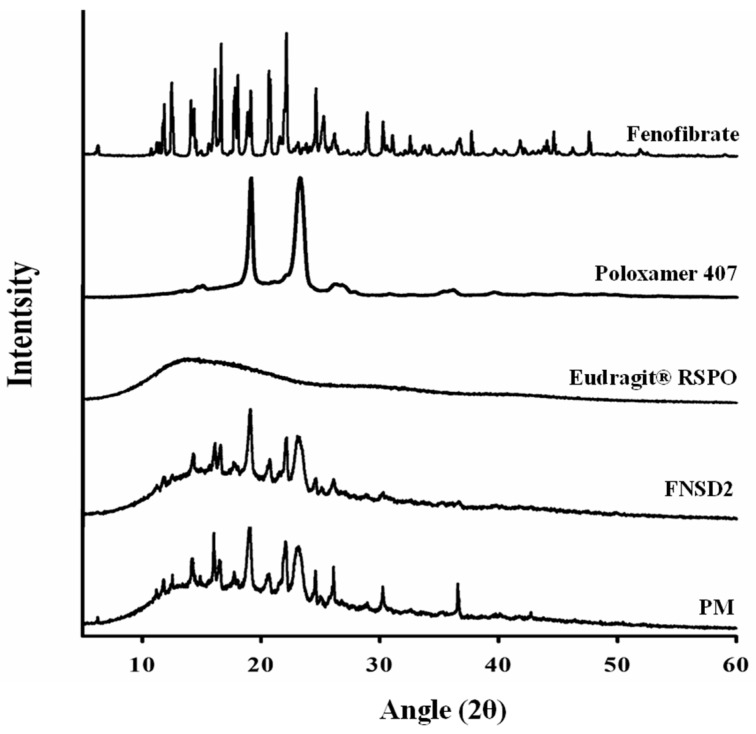
X-ray diffraction (XRD) patterns of fenofibrate, poloxamer 407, Eudragit^®^ RSPO, physical mixture (PM), and the FNSD2 formulation.

For more clarification, XRD analysis was conducted to evaluate the crystalline state of drugs in FNSD2. As shown in Figure 5, the diffraction profile of pure drug indicated its crystalline nature, displaying many distinct peaks at 2*θ* angles in the range of 5–50° in concordance with those in previous reports [37]. While Eudragit^®^ RSPO is amorphous, poloxamer 407 is in a crystalline state, exhibiting distinct peaks at 19.13° and 23.21° (Figure 5). Although the intensity was decreased due to the dilution effect by excipients, both PM and FNSD2 showed diffraction patterns similar to those of the pure drug, confirming the crystalline state of the drugs. These results suggest that the enhanced drug dissolution via FNSD2 might be due to the decreased particle size and the surface-active properties of poloxamer 407 rather than amorphization [38].

### 3.4. Pharmacokinetics in Rats

The pharmacokinetic profiles of fenofibrate after oral administration of pure drug, PM, and the FNSD2 formulation were evaluated in rats. Since fenofibrate is rapidly converted to its active metabolite, fenofibric acid [39], the oral pharmacokinetics of fenofibrate were evaluated based on the plasma concentration of fenofibric acid.

**Figure 6:**
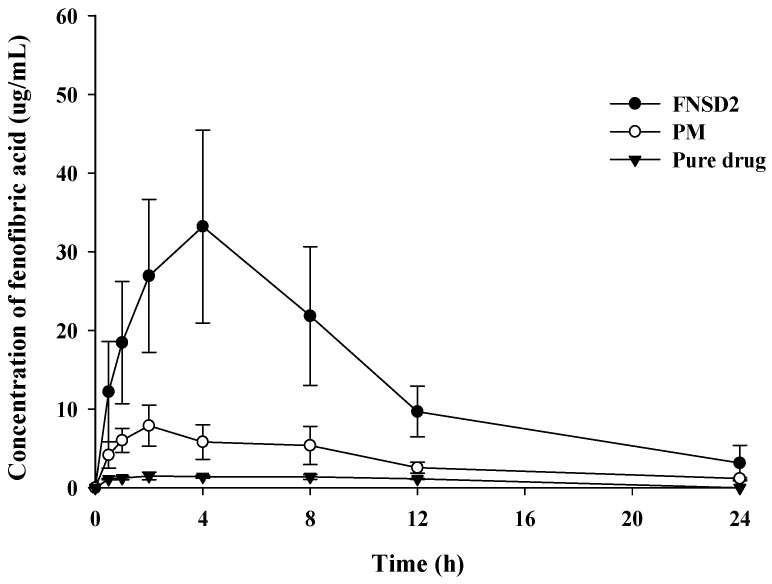
Pharmacokinetic profiles of fenofibric acid (an active metabolite of fenofibrate) following an oral administration of fenofibrate in different formulations to rats (Mean ± SD, n = 6). The dose is equivalent to 20 mg/kg of fenofibrate.

As summarized in Table 4 and Figure 6, the oral exposure to the pure drug was low, resulting in a C_max_ of 1.64 μg/mL and an AUC of 15.7 μg·h/mL. In addition, T_max_ was 3.4 h, implying the slow drug dissolution and absorption of poorly soluble drugs. These results are comparable to previous reports [40]. Compared to the pure drug, PM enhanced drug absorption, increasing C_max_ and AUC by 4.9−5.4 folds. This may be explained by the solubilization effect of poloxamer 407, a nonionic surfactant. In the presence of poloxamer 407, the enhanced drug solubility may promote in vivo drug dissolution and subsequent intestinal absorption, improving systemic drug exposure. Compared to pure drug and PM, the FNSD2 formulation was much more effective in improving systemic drug exposure for a longer period (Figure 6). FNSD2 achieved a C_max_ of 33.2 ± 12.3 μg/mL and an AUC of 344 ± 125 μg·h/mL, representing a 22-fold increase in oral bioavailability of fenofibrate. Simultaneously, it showed gradual drug absorption with delayed T_max_ and maintained the systemic exposure higher than the C_max_ of the pure drug over 24 h. These results were attributed to the sustained but enhanced drug dissolution of FNSD2. BCS II drugs such as fenofibrate have low solubility and high permeability, thereby exhibiting dissolution-limited drug absorption [41]. Accordingly, the enhanced drug solubility and dissolution via FNSD2 led to the significantly improved oral bioavailability of fenofibrate (Table 4 and Figure 6). In the similar experiments previously reported, the increase in oral bioavailability of fenofibrate was much lower than that of FNSD2. For example, Yousaf et al. [22] developed a gelatin nanocapsule of fenofibrate, which improved the oral bioavailability of fenofibrate by 5.5-fold. Ahn et al. [42] prepared the amorphous SD of fenofibrate using a supercritical anti-solvent process, which enhanced the oral bioavailability of fenofibrate by approximately 11-fold compared to the pure drug [42]. Yang et al. [43] also developed an omega-3 phospholipid-based SD of fenofibrate, improving the oral bioavailability by approximately 7-fold. Although the direct comparison to the literature data may not be appropriate due to the different experimental conditions, FNSD2 appeared to be much more effective in improving the oral bioavailability of fenofibrate via simultaneous enhancement of the extent and duration of drug exposure.

Overall, the results support the FNSD2 formulation as an effective means of delivering poorly soluble fenofibrate, offering enhanced bioavailability and sustained-release characteristics that could provide substantial clinical benefits in managing hyperlipidemia.

## 4. Conclusions

The sustained-release solid dispersion of fenofibrate, using a combination of hydrophilic and hydrophobic polymers (poloxamer 407 and Eudragit^®^ RSPO), was optimized in the FNSD2 formulation with a 1:1:4 (*w*/*w*/*w*) ratio. FNSD2 demonstrated suitable dissolution profiles for sustained drug release while significantly enhancing drug dissolution of poorly soluble fenofibrate. Pharmacokinetic evaluations in rats indicated that FNSD2 improves oral bioavailability and extends the duration of drug exposure, providing an effective strategy for oral delivery of fenofibrate.

## Figures and Tables

**Table 1:** Composition of fenofibrate-loaded solid dispersions.

Formulation	Ratio (*w*/*w*/*w*)		
	Fenofibrate	Poloxamer 407	Eudragit^®^ RSPO
FNSD1	1	0.5	4
FNSD2	1	1	4
FNSD3	1	2	4
FNSD4	1	1	2
FNSD5	1	1	8
FNSD6	1	1	10

**Table 2:** Aqueous solubility of fenofibrate from different formulations (Mean ± SD, n = 3).

Formulation	Solubility (μg/mL)
Fenofibrate (untreated powder)	0.21 ± 0.05
FNSD1	0.36 ± 0.09
FNSD2	3.65 ± 0.63
FNSD3	12.0 ± 0.47
PM1	0.24 ± 0.05
PM2	0.33 ± 0.04
PM3	1.00 ± 0.11

PM1, PM2, and PM3 are physical mixtures corresponding to FNSD1, FNSD2, and FNSD3, respectively.

**Table 3:** Release kinetic parameters of the FNSD2 formulation.

Formulation	Zero Order		Frist Order		Higuchi		Korsmeyer-Peppas		
	r^2^	k_1_	r^2^	k_2_	r^2^	k_3_	r^2^	k_4_	n
FNSD2	0.7953	4.8	0.912	−0.1081	0.9263	20.7	0.9552	0.4184	0.4072

**Table 4:** Pharmacokinetic parameters of fenofibric acid after oral administration of fenofibrate in different formulations to rats (Mean ± SD, n = 6). The dose is equivalent to 20 mg/kg of fenofibrate.

Parameter	Pure Drug	PM	FNSD 2
C_max_ (µg/mL)	1.64 ± 0.37	8.09 ± 2.34	33.2 ± 12.3 *
T_max_ (h)	3.4 ± 2.3	1.8 ± 0.4	4.0 ± 0.0
AUC (µg∙h/mL)	15.7 ± 2.23	84.9 ± 19.9	344 ± 125 *

* *p* < 0.05, statistically significant difference in comparison with the pure drug.

## Data Availability

Data are contained within the article.
